# Association between anhedonia severity and clinical, humanistic, and economic outcomes among US adults with major depressive disorder

**DOI:** 10.1093/ijnp/pyaf048

**Published:** 2025-07-02

**Authors:** Hrishikesh Kale, M Janelle Cambron-Mellott, Tiina Drissen, Kacper Perkowski, Kristen King-Concialdi, Manish K Jha

**Affiliations:** Scientific Evidence & Policy Research (SEPR), Johnson & Johnson, Titusville, NJ, United States; Real World Evidence, Oracle Life Sciences, Austin, TX, United States; US Medical Affairs, Johnson & Johnson, Titusville, NJ, United States; Real World Evidence, Oracle Life Sciences, Austin, TX, United States; Real World Evidence, Oracle Life Sciences, Austin, TX, United States; Center for Depression Research and Clinical Care, Department of Psychiatry, University of Texas Southwestern Medical Center, Dallas, TX, United States

**Keywords:** anhedonia, clinical burden, economic burden, health-related quality of life, major depressive disorder

## Abstract

**Background:**

Anhedonia is a key symptom and part of the diagnostic criterion of major depressive disorder (MDD). However, the relationship between anhedonia severity and the clinical, humanistic, and economic burden among patients with MDD is poorly understood.

**Methods:**

Adults diagnosed with depression were identified from the 2022 US National Health and Wellness Survey (NHWS). Participants with depression were recontacted to participate in an online cross-sectional survey to collect data on anhedonia, using the Snaith-Hamilton Pleasure Scale (SHAPS). Multivariable analyses assessed the association of SHAPS score with health-related outcomes, while controlling for age, sex, race, comorbidity burden, and insurance status. The SHAPS (score range: 14 to 56) assesses the ability to experience pleasure, with higher scores indicating greater levels of anhedonia.

**Results:**

Of the 8270 NHWS respondents with depression who met inclusion criteria, 665 completed the recontact survey (mean age, 58.4 years; female, 78.3%). Mean SHAPS score was 25.4 (range, 14–47). After adjustments for covariates, higher SHAPS scores were significantly (all *P* <.05) associated with higher levels of depression (β = 0.211) and anxiety (β = 0.126), poorer mental (β = −0.339) and physical health-related quality of life (β = −0.178), greater impairment while working [rate ratio (RR) = 1.02], and higher direct medical costs (RR = 1.02).

**Conclusions:**

In adults with depression, higher levels of anhedonia were associated with greater clinical, humanistic, and economic burden. These results highlight the need for targeted treatments to help patients with MDD with prominent anhedonia attain improved clinical, humanistic, and work productivity outcomes.

Significance Statement In this study, we examined the relationship between anhedonia (lack of interest, enjoyment or pleasure from life’s experiences), which is a core symptom of major depressive disorder (MDD) and clinical, humanistic and economic outcomes in patients with MDD. In a large sample of MDD patients from National Health & Well Survey, higher levels of anhedonia were linked with greater depression and anxiety severity, poorer health-related quality of life, greater impairment while working and while performing non-work-related activities, and higher direct medical costs among patients with MDD. This study highlights the significant unmet need associated with anhedonia, importance of anhedonia identification and a need for targeted treatments to improve outcomes in adults with MDD.

## INTRODUCTION

Major depressive disorder (MDD) is a leading cause of disability globally.^1^ In 2020, about 21.0 million (8.3%) of the United States (US) adult population had experienced at least one major depressive episode.^2^ The estimated lifetime prevalence of MDD in the US is 20.6%, the second most reported cause of disability in adults,^3^^,^^4^ and is associated with impaired health-related quality of life (HRQoL), increased healthcare resource utilization (HCRU) and associated costs.^5–7^ Anhedonia is a core symptom and part of the diagnostic criterion of MDD.^8^ According to the Diagnostic and Statistical Manual of Mental Disorders, Fifth Edition Text Revision, anhedonia is defined as “markedly diminished interest or pleasure in all, or almost all activities most of the day, nearly every day.”^3^ The prevalence of anhedonia in patients with MDD ranges from 37% to 70% and varies depending on the study and the population being studied.^8–11^

The clinical burden associated with anhedonia and the impact it has on patients with MDD is substantial and often under-recognized. Anhedonia negatively impacts psychosocial functioning and has been associated with longer times to remission and a reduced number of depression-free days.^12^^,^^13^ In addition, anhedonia predicts poor antidepressant treatment outcomes^14^ and is associated with reduced HRQoL and increased functional impairment.^15^ Furthermore, it has been shown that higher level of anhedonia is often associated with increased risk of suicidal behavior.^8^^,^^16^

While some studies have assessed the association of anhedonia with HRQoL,^15^^,^^17^ employment status,^18^ social interactions, ^19^ and psychosocial functioning,^13^ they were limited by small sample sizes and to non-US populations. There has been limited research on the burden associated with severity of anhedonia in patients with MDD. Moreover, studies examining the relationship of HCRU and associated costs with anhedonia in MDD has not been explored adequately. Therefore, the objective of this study was to quantify the clinical, humanistic, and economic burden associated with the severity of anhedonia among adults in the US who self-reported physician diagnosed MDD, using a nationally representative data source. In addition, this study afforded the opportunity to expand on prior work by utilizing two measures of anhedonia: (1) the more widely used Snaith-Hamilton Pleasure Scale (SHAPS), which measures one aspect of anhedonia, namely consummatory pleasure^20^ and (2) the Dimensional Anhedonia Rating Scale (DARS), which measures multiple dimensions of anhedonia, including interest/desire, motivation, effort, and consummatory pleasure^21^ and represents a more comprehensive measure of anhedonia.

## METHODS

### Study Design and Data Source

This retrospective, cross-sectional study used real-world data from the 2022 US National Health and Wellness Survey (NHWS; May–September 2022) and an additional survey administered to depression-diagnosed participants who completed the 2022 NHWS. The NHWS is a nationally representative, self-reported, online survey conducted annually among the general adult population in the US (aged ≥18 years; *N* = ~75 000). Potential participants for the survey were recruited through an existing, general-purpose (not healthcare-specific) web-based consumer research panel. The research panel members were recruited through opt-in e-mails, co-registration with panel partners, e-newsletter campaigns, banner placements, and affiliate networks. The NHWS used a quota sampling procedure with strata for sex, race/ethnicity, and age, to ensure that the demographic composition of the NHWS sample was representative of the adult population in the US. NHWS participants with depression meeting inclusion criteria were recontacted and invited to participate in an online cross-sectional survey to assess anhedonia. Data collection occurred during August–October 2022. Participants took approximately 15 minutes to complete the survey, and those who completed the full survey received fair market value compensation for their time.

The study was conducted per the Declaration of Helsinki and Good Epidemiological Practices recommended by the International Society of Pharmacoeconomics and Outcomes Research. The NHWS protocol and questionnaire and the recontact protocol and questionnaire were granted exemption status from Pearl Institutional Review Board (Indianapolis, IN) in accordance with Food and Drug Administration 21 CFR 56.104 and 45 CRF 46.104 (b) (2). Informed consent was obtained from all NHWS and recontact survey participants.

### Study Population

Participants who completed the 2022 US NHWS, were aged ≥18 years, resided in the US, self-reported a physician diagnosis of depression or current prescription use for treatment of depression, self-reported experiencing depression in the past 12 months, and completed the recontact study were included. Participants were excluded if they self-reported ever experiencing bipolar disorder or schizophrenia. We did not exclude participants who reported having any other psychiatric comorbidities.

### Measures

Sociodemographic information collected were age, sex, race, ethnicity, marital status, employment, education, and insurance status. Health characteristics included body mass index, smoking status, exercise behavior, and the Charlson Comorbidity Index (CCI; greater scores indicating greater comorbid burden on the patient).^22^ The CCI measure included the following comorbidities: human immunodeficiency virus/acquired immunodeficiency syndrome, any malignancy (including lymphoma and leukemia), metastatic tumor, renal disease, hemiplegia, mild liver disease, severe liver disease, rheumatologic disease, chronic pulmonary disease, dementia, congestive heart failure, and diabetes with end organ damage.^22^ Other mental health/psychiatric conditions, such as anxiety were also analysed.

#### Anhedonia Severity

Anhedonia severity was assessed by SHAPS,^20^ which assesses the ability to experience pleasure in four domains: interests/pastimes, social interaction, sensory experience, and food/drink. Participants rated 14-items on a 4-point scale from 1 (strongly disagree) to 4 (strongly agree); items were summed to form a measure of severity (range: 14–56). Higher scores indicate a greater level of anhedonia.^23^

DARS was also used to measure anhedonia severity. DARS is a 17-item instrument that assesses the desire, motivation, effort, and consummatory pleasure across four domains: hobbies, food/drinks, social activities, and sensory experience. Participants provided responses using a 5-point Likert scale from 0 (not at all) to 4 (very much). All items were summed to form a total score (range: 0–68). Higher scores indicate greater levels of motivation, effort, and pleasure (i.e., less anhedonia).^21^

#### Clinical Burden

Depression severity was measured using the 9-item Patient Health Questionnaire (PHQ-9).^24^ The PHQ-9 measures frequency of depression symptoms experienced in the past 2 weeks, with items rated on a 4-point scale (0 = not at all to 3 = nearly every day). Items were summed to form a total score (range: 0–27). Higher scores indicate a greater level of depression.

The 7-item Generalized Anxiety Disorder (GAD-7) questionnaire measured the degree of anxiety symptoms experienced over the past 2 weeks.^25^ Participants rated score on seven symptoms of anxiety which they experienced using a 4-point scale ranging from 0 (not at all) to 3 (nearly every day). Scores are summed to form a total score (range: 0–21), with higher scores indicating greater disease severity.

#### Humanistic Burden

HRQoL was assessed using the RAND-36 health survey; a multipurpose, generic HRQoL instrument,^26^ which is designed to report two summary scores: Mental Health Composite (MHC) and Physical Health Composite (PHC) scores (range: 0–100). The scores are normed to a mean of 50 with a standard deviation (SD) of 10 for the general population; higher scores indicate better HRQoL. Additionally, health status and self-rated health was calculated using the 5-level EQ-5D version (EQ-5D-5L).^27^ The EQ-5D-5L consists of a descriptive system (EQ-5D), which is used to compute EQ-5D utility index scores (range: 0–1, with 0 indicating health state equivalent to death and 1 indicating perfect health) and the visual analog scale (EQ VAS; range: 0–100) indicates the participant’s self-rated health, with 0 equivalent to worst imaginable health state and 100 indicating the best imaginable health state.^27^

#### Economic Burden

Impairments in work productivity and activity impairment due to one’s health were assessed using the Work Productivity and Activity Impairment (WPAI) questionnaire, a 6-item validated instrument that assessed four domains with a 1-week recall period: absenteeism (percent work time missed due to health problems), presenteeism (percent impairment while working due to health problems), overall work productivity impairment, and activity impairment.^28^ Only participants who reported being full-time, part-time, or self-employed provided data for absenteeism, presenteeism, and overall work impairment. All respondents provided data for activity impairment. Higher scores indicate greater impairment.

HCRU was measured based on the number of visits to traditional healthcare providers (HCP), psychiatrists, psychologists/therapists, and emergency rooms (ER) in the past 6 months, and the number of hospitalizations in the past 6 months, as self-reported by the participants.

Direct costs were estimated by annualizing the number of HCP visits, ER visits, and hospitalizations, multiplied by the unit cost for each type of visit obtained from the Medical Expenditure Panel Survey data^29^ and then inflated to 2020 medical care costs.^30^

### Statistical Analysis

Descriptive statistics were reported using mean and SD for continuous variables and counts and percentages for categorical variables. Pearson’s correlation analysis was used to examine the bivariate association between anhedonia severity (SHAPS and DARS) and depression (PHQ-9). In multivariable analyses, generalized linear models (GLMs) were used to assess outcomes as a function of level of anhedonia (SHAPS and DARS) with clinical, humanistic, and economic outcomes after adjusting for age, sex, race, CCI, and insurance status. GLMs with identity link function were run for clinical (PHQ-9, GAD-7) and HRQoL outcomes. GLMs specifying negative binomial distribution and log-link were run for HCRU and WPAI outcomes, and GLMs with log link functions were run for economic outcomes. Parameter estimates (β) with standard errors were reported for all outcomes. In addition, adjusted rate ratios (RR) with 95% confidence intervals (CIs) were reported for HCRU, WPAI, and economic outcomes. Two-tailed tests were considered statistically significant when *P* < .05. Adjusted means for outcome measures across the range of SHAPS and DARS scores were predicted and plotted based on corresponding multivariable GLM outputs. Parameter estimates and RRs were also adjusted to account for a 1-SD increase in anhedonia (1-SD increase in SHAPS scores, 1-SD decrease in DARS scores).

## RESULTS

Of the 8910 NHWS respondents with depression who met the eligibility criteria, 665 completed the recontact survey and were included in the final analysis sample (Figure S1). Table S1 shows key sociodemographic characteristics of the NHWS respondents meeting eligibility criteria and the final analysis sample.

The mean (SD) age of the respondents was 58.4 (13.4) years, with majority being female (78.3%), White (86.2%), and non-Hispanic (94.7%). Anxiety was observed as the most comorbid diagnosed mental health condition (68.6%). The mean (range) SHAPS score among the sample was 25.4 (14–47), and the mean (range) DARS score was 52.6 (2–68) (Table 1). In correlation analysis, higher anhedonia severity was associated with greater depression severity, as measured by SHAPS score, (*r* = 0.24, *P* <.001) and by DARS score (*r* = −0.31, *P* <.001) (Table S2).

**Table 1:** Sociodemographic, health and clinical characteristics of MDD respondents.

**Characteristic**	**MDD (*n* = 665)**
**Gender, n (%)**	
** Male**	144 (21.7)
** Female**	521 (78.3)
**Age, years, mean (SD)**	58.4 (13.4)
**Age category, n (%)**	
** 18 to <25**	20 (3.0)
** 25 to <35**	31 (4.7)
** 35 to <45**	38 (5.7)
** 45 to <55**	114 (17.1)
** 55 to <65**	210 (31.6)
** 65 and older**	252 (37.9)
**Race, n (%)**	
** White**	573 (86.2)
** Black/African American**	50 (7.5)
** Asian**	11 (1.7)
** Other**	31 (4.7)
**Ethnicity, n (%)**	
** Hispanic**	35 (5.3)
** Non-Hispanic**	630 (94.7)
**Marital status, n (%)**	
** Married/living with a partner**	304 (45.7)
** Single/divorced/ separated/widowed**	361 (54.3)
** Education, college degree or higher, n (%)**	249 (37.5)
**Employment, n (%)**	
** Employed^a^**	232 (34.9)
** Retired**	248 (37.3)
** Short-/long-term disability**	73 (11.0)
** Homemaker or student**	53 (8.0)
** Not employed**	59 (8.9)
**Insurance type, n (%)**	
** Commercially insured**	225 (33.8)
** Medicaid**	106 (15.9)
** Medicare**	272 (40.9)
** Other type of insurance**	26 (3.9)
** BMI, mean (SD)**	31.4 (8.1)
**Smoking status, n (%)**	
** Current smoker**	137 (20.6)
** Former smoker**	215 (32.3)
** Never smoker**	313 (47.1)
** Days exercising in the past month** ^**b**^ **, mean (SD)**	5.6 (8.3)
** CCI score, mean (SD)**	1.20 (2.0)
** PHQ-9 score, mean (SD)**	10.0 (6.6)
**SHAPS score**	
** Mean (SD)**	25.4 (6.3)
** Median (range)**	25 (14–47)
**DARS score**	
** Mean (SD)**	52.6 (13.5)
** Median (range)**	55 (2–68)
**Diagnosed mental health comorbidities, n (%)**	
** Anxiety/Generalized Anxiety Disorder**	456 (68.6)
** Attention deficit disorder**	38 (5.7)
** Attention deficit hyperactivity disorder**	16 (2.4)
** Obsessive compulsive disorder**	54 (8.1)
** Panic disorder**	83 (12.5)
** Phobias**	13 (2.0)
** Post-traumatic stress disorder**	104 (15.6)
** Social anxiety disorder**	103 (15.5)
** Currently on prescription medication for depression, n (%)**	434 (65.3)
**By drug class**	
** Selective serotonin reuptake inhibitor (SSRI)**	261 (60.1)
** Serotonin and norepinephrine reuptake inhibitor (SNRI)**	112 (25.8)
** Norepinephrine and dopamine reuptake inhibitor (NDRI)**	110 (25.3)
** Serotonin modulator**	42 (9.7)
** Atypical antipsychotic**	36 (8.3)
** Tricyclic antidepressant (TCA)**	22 (5.1)
** Tetracyclic antidepressant (TeCA)**	21 (4.8)
** Mood stabilizer**	8 (1.8)
** Monoamine oxidase inhibitors (MAOI)**	1 (0.2)

^a^Employed full-time, part-time, or self-employed

^b^Number of days in the past month of ≥20 minutes of vigorous exercise

Abbreviations: BMI, body mass index; CCI, Charlson comorbidity index; MDD, major depressive disorder; PHQ-9, 9-item Patient Health Questionnaire; SD, standard deviation; SHAPS, Snaith-Hamilton Pleasure Scale.

### Clinical, Humanistic, and Economic Burden of Anhedonia Severity, as Measured by SHAPS Score

**Figure 1:**
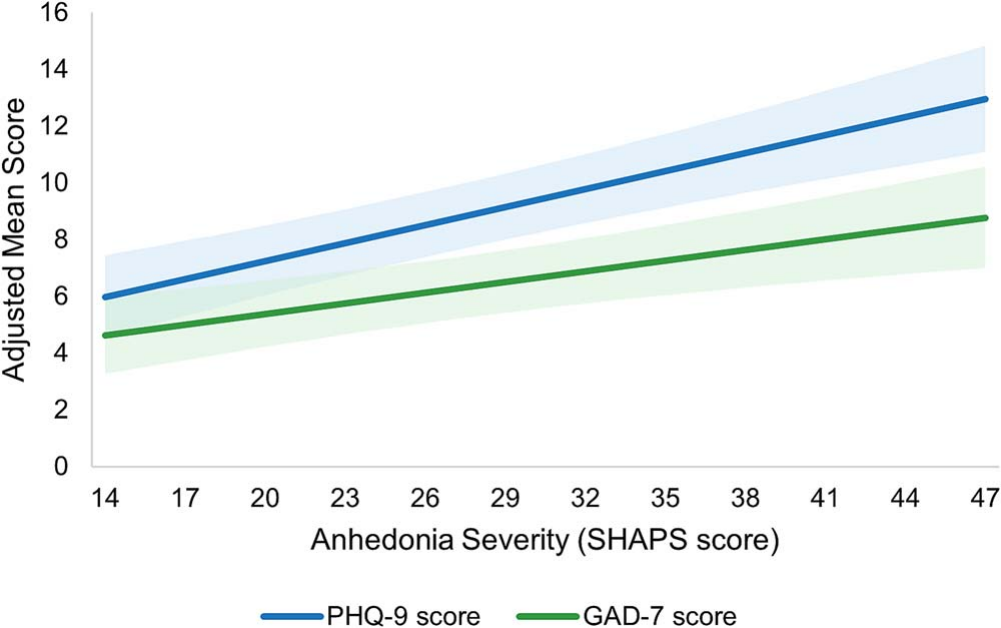
Adjusted clinical outcomes by anhedonia severity: depression and anxiety. Note: Reference groups—age: 58.35 years, gender: male, race: White, insurance: commercial, CCI: 1.20. Higher SHAPS scores indicate greater anhedonia; shading represents 95%CI. CCI, Charlson comorbidity index; CI, confidence intervals; GAD-7, 7-item Generalized Anxiety Disorder scale; PHQ-9, 9-item Patient Health Questionnaire; SHAPS, Snaith-Hamilton Pleasure Scale.

In adjusted analysis, higher anhedonia severity (as measured by SHAPS) was associated with greater depression severity (*β* = 0.211, *P* <.001) and greater anxiety severity (*β* = 0.126, *P* <.001; Figure 1 and Table 2).

**Table 2:** Multivariable analyses of association of MDD-ANH severity with clinical, humanistic, and economic outcomes: SHAPS score

					**1-SD increase** **(6.25 points)**	
**Outcomes**	**N**	***β* (SE)**	**RR** ^**a**^ **(95% Cl)**	***P*-value**	***β***	**RR** ^**a**^
**Screening tools** ^**b**^						
** PHQ-9 score**	665	0.211 (0.037)	–	**<0.001**	1.319	–
** GAD-7 score**	665	0.126 (0.035)	–	**<0.001**	0.786	–
**HCRU in past 6 months** ^**c**^						
** HCP visits**	665	0.008 (0.006)	1.01 (0.10-1.02)	0.176	0.051	1.05
** ER visits**	665	0.016 (0.014)	1.02 (0.99-1.05)	0.262	0.101	1.11
** Hospitalizations**	665	0.015 (0.020)	1.01 (0.98-1.06)	0.456	0.091	1.10
** Psychiatrist visits**	665	−0.029 (0.027)	0.97 (0.92-1.02)	0.280	−0.179	0.84
** Psychologist/therapist visits**	665	0.013 (0.030)	1.02 (0.96-1.07)	0.651	0.084	1.09
**HRQoL** ^**b**^						
** RAND MHC score**	665	−0.339 (0.066)	–	**<0.001**	−2.116	–
** RAND PHC score**	665	−0.178 (0.068)	–	**0.009**	−1.113	–
** EQ-5D Index score**	665	−0.004 (0.001)	–	**<0.001**	−0.025	–
** EQ VAS score**	665	−0.459 (0.159)	–	**0.004**	−2.868	–
**WPAI, (mean %)** ^**c**^						
** Absenteeism**	209	0.023 (0.038)	1.02 (0.95-1.10)	0.545	0.143	1.15
** Presenteeism**	203	0.016 (0.002)	1.02 (1.01-1.02)	**<0.001**	0.101	1.11
** Work Productivity Impairment**	203	0.013 (0.016)	1.01 (0.98-1.05)	0.420	0.082	1.09
** Activity Impairment^**d**^**	665	0.010 (0.001)	1.01 (1.01-1.01)	**<0.001**	0.060	1.06
**Economic**						
** Total direct medical costs^**e**^**	665	0.023 (0.011)	1.02 (1.00-1.04)	**0.033**	0.141	1.15
** Office visit costs^**e**^**	665	0.016 (0.007)	1.02 (1.00-1.03)	**0.019**	0.102	1.11
** ER visit costs^**e**^**	665	0.013 (0.010)	1.01 (0.99-1.03)	0.192	0.082	1.09
** Inpatient costs^**e**^**	665	0.028 (0.016)	1.03 (1.00-1.06)	0.077	0.176	1.19

Note: controlled for age, sex, race, CCI, and insurance. Bolded *P*-values denote statistical significance.

a exp(β) is not presented for HRQoL or economic items. Exp(β), the rate ratio, is presented for all other items.

b GLM w/ Identity link; Interpretation: For each 1-point/1-SD increase in SHAPS score < the outcome> changes by an average of <β>, keeping other predictors constant.

c GLM w/ Negative Binomial distribution; Interpretation: For each 1-point/1-SD increase in SHAPS score < the outcome> is <exp(β) > times higher, keeping other predictors constant.

d Race control variable collapsed to White/Non-White due to convergence issues.

e GLM w/ Log link; Interpretation: For each 1-point/1-SD increase in SHAPS score < the outcome> is exp(β) > times higher, keeping other predictors constant.

Abbreviation: ANH, anhedonia; CCI, Charlson Comorbidity Index; CI, confidence interval; ER, emergency room; GAD-7, 7-item Generalized Anxiety Disorder; GLM, generalized linear model; HCP, healthcare provider; HRCU, healthcare resource utilization; HRQoL, health-related quality of life; MDD, major depressive disorder; MHC, Mental Health Composite; PHC, Physical Health Composite; PHQ-9, 9-item Patient Health Questionnaire; RR, rate ratio; SD, standard deviation; SE, standard error; SHAPS, Snaith-Hamilton Pleasure Scale; VAS, visual analogue scale; WPAI, work productivity and activity impairment.

**Figure 2:**
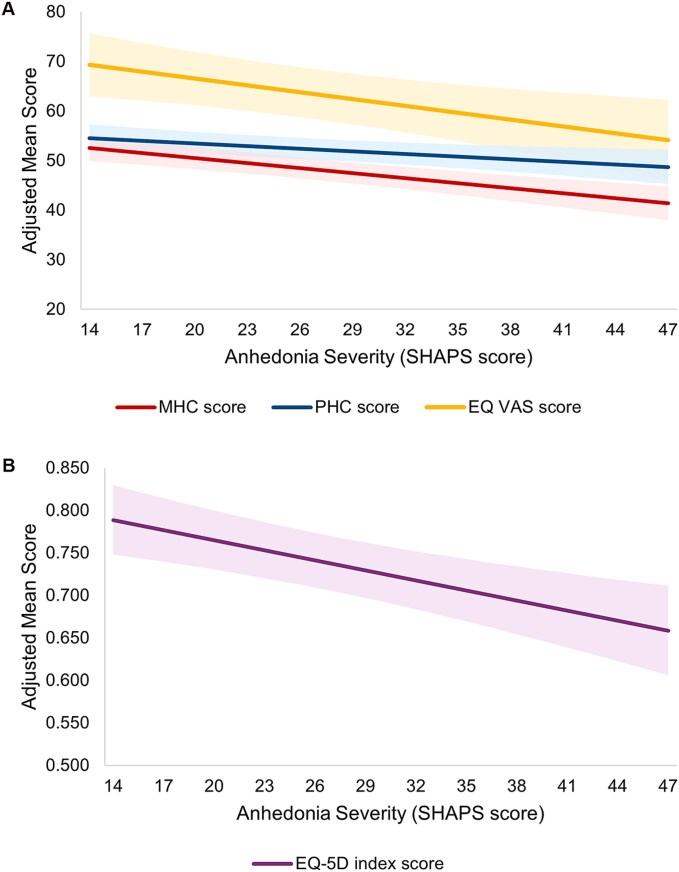
Adjusted humanistic outcomes by anhedonia severity: HRQoL. A) RAND-36 and EQ VAS scores. B) EQ-5D index scores. Note: Reference groups—age: 58.35 years, gender: male, race: White, insurance: commercial, CCI: 1.20. Higher SHAPS scores indicate greater anhedonia; shading represents 95% CI. CCI, Charlson comorbidity index; CI, confidence interval; HRQoL, health-related quality of life; MHC, Mental Health Composite; PHC, Physical Health Composite; SHAPS, Snaith-Hamilton Pleasure Scale; VAS, visual analogue scale.

Higher severity of anhedonia (i.e., higher SHAPS scores) was associated with worse HRQoL, including poorer mental function (*β* = −0.339, *P* <.001), poorer physical function (*β* = −0.178, *P* = .009), lower EQ-5D index scores (*β* = −0.004, *P* < .001), and lower EQ VAS scores (*β* = −0.459, *P* = .004) (Figure 2 and Table 2).

**Figure 3:**
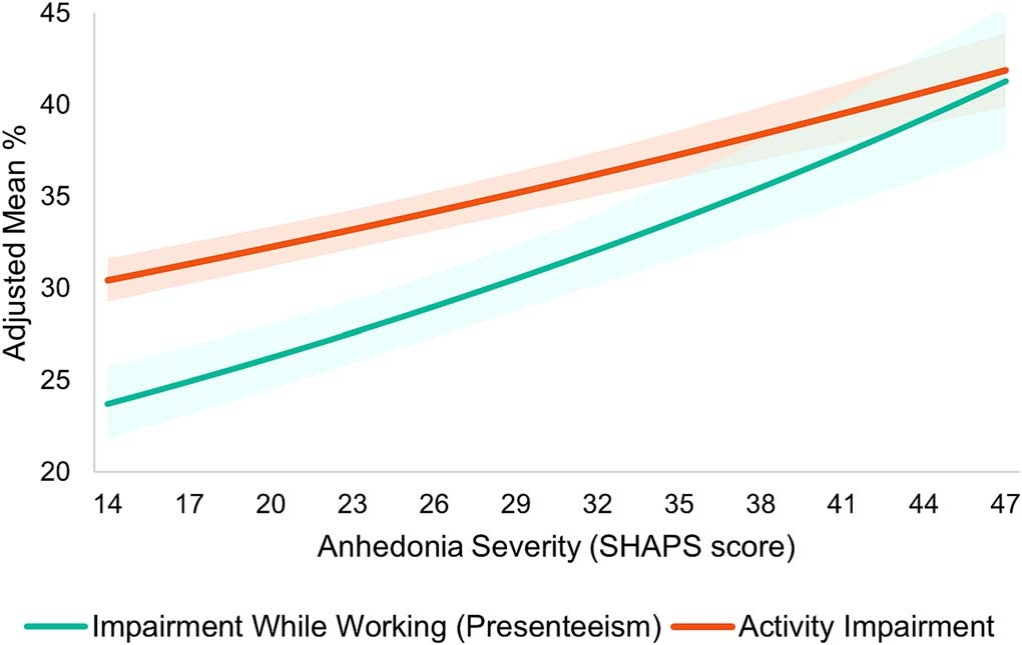
Adjusted economic outcomes by anhedonia severity: WPAI. Note: Reference groups—age: 58.35 years, gender: male, race: White, insurance: commercial, CCI: 1.20. Higher SHAPS scores indicate greater anhedonia; shading represents 95% CI. CCI, Charlson comorbidity index; CI, confidence interval; SHAPS, Snaith-Hamilton Pleasure Scale; WPAI, work productivity and activity impairment.

Higher anhedonia severity as measured by higher SHAPS score was associated with greater impairment while working (presenteeism, RR = 1.02, *P* < .001) and greater activity impairment (RR = 1.01, *P* < .001; Figure 3 and Table 2).

**Figure 4:**
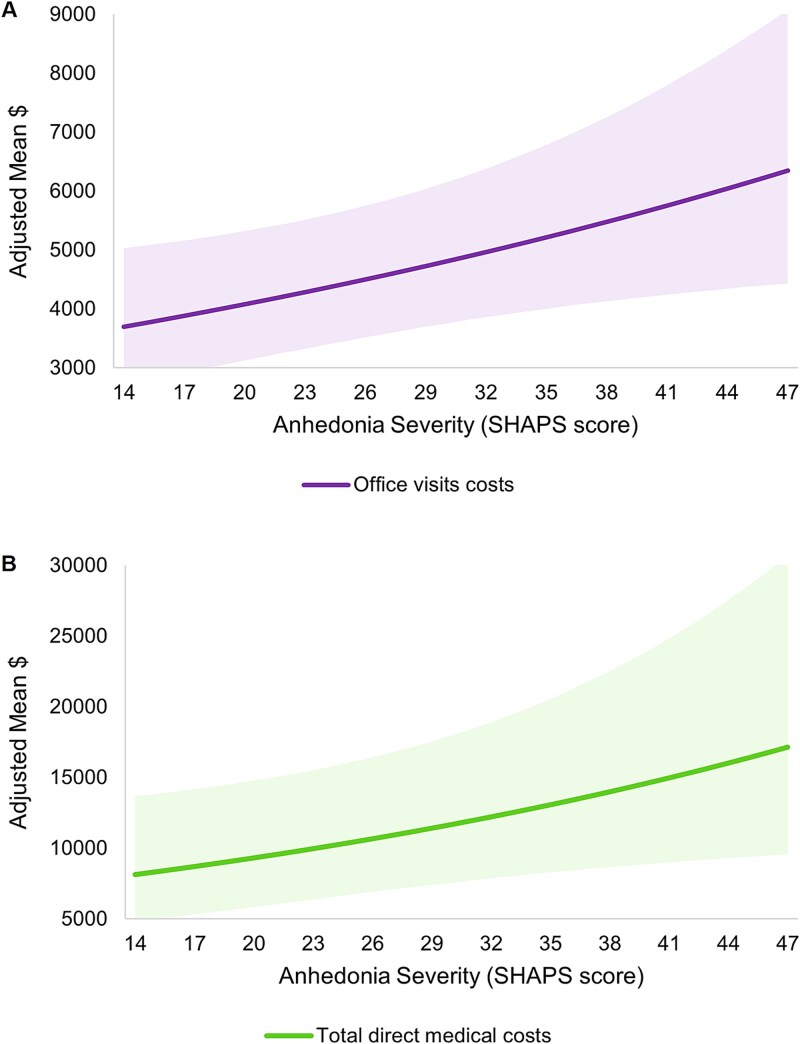
Adjusted economic outcomes by anhedonia severity: direct medical costs. A) Office visits costs. B) Total direct medical costs. Note: Reference groups—age: 58.35 years, gender: male, race: White, insurance: commercial, CCI: 1.20. higher SHAPS scores indicate greater anhedonia; shading represents 95% CI. CCI, Charlson comorbidity index; CI, confidence interval; SHAPS, Snaith-Hamilton Pleasure Scale.

Although there were no statistically significant associations between anhedonia and HCRU in the past 6 months (Table 2), higher anhedonia severity (i.e., higher SHAPS scores) was associated with higher office visit costs (RR = 1.02, *P* = .019) and higher total direct medical costs (RR = 1.02, *P* = .033) (Figure 4 and Table 2).

### Clinical, Humanistic, and Economic Burden of Anhedonia Severity, as Measured by DARS Score

Similar to the results using SHAPS score as the measure of anhedonia, higher severity of anhedonia (i.e., lower DARS scores) was associated with greater depression severity (*β* = −0.132, *P* < .001), greater anxiety severity (*β* = −0.056, *P* < .001), poorer mental function (*β* = 0.144, *P* < .001), lower EQ-5D index scores (*β* = 0.002, *P* < .001), greater impairment while working (presenteeism, RR = 0.995, *P* < .001), greater activity impairment (RR = 0.996, *P* < .001), and higher outpatient office visits costs (RR = 0.99, *P* < .001). In contrast to the SHAPS results, higher anhedonia (as measured by lower DARS scores) was significantly associated with more HCP visits (RR = 0.99, *P* = .002) and more psychologist/therapist visits (RR = 0.97, *P* = .015) in the past 6 months (Table S3 and Figures S2-S6).

## DISCUSSION

This retrospective, cross-sectional, real-world study assessed clinical, humanistic, and economic burden associated with anhedonia severity among adults with MDD in the US. In this study, greater anhedonia severity was associated with greater depression and anxiety severity, poorer HRQoL, greater impairment while working and while performing non-work-related activities, and higher direct medical costs among patients with MDD.

In the present study, higher levels of anhedonia (as measured by SHAPS and DARS) were significantly associated with greater depression and anxiety severity**.** Our study results corroborate with prior findings, wherein a study by Kaviani et al. showed a positive correlation between anhedonia and depression.^31^ In another study, positive correlations between anhedonia and depression were observed in a subset of depressed patients, indicating strong psychopathological relations with negative and depressive symptoms.^10^ Moreover, anhedonia was found to be the mediating risk factor between anxiety and depression cross-sectionally and over time.^32^

Further, higher severity of anhedonia (as measured by SHAPS) was associated with reduced HRQoL, including lower scores on mental function and physical function, EQ-5D, and EQ VAS after adjusting for covariates (age, sex, race, CCI, and insurance status). Consistently, higher severity of anhedonia based on DARS measure were associated with poorer mental function and lower EQ-5D scores. Our findings are in agreement with previous studies that reported decreased HRQoL in terms of poor mental health and physical health, reduced life enjoyment and satisfaction.^15^^,^^17^ In a study by Whitton et al, anhedonia was associated with worse HRQoL cross-sectionally as well as longitudinally among individuals with depression. Further, anhedonia was found to be a predictor of mental HRQoL, life enjoyment and satisfaction, at baseline and 3- and 6-month follow-up after controlling for non-anhedonic symptoms of depression and anxiety.^17^ In a recent systematic review and meta-analysis, anhedonia was negatively associated with self-reported HRQoL and functional outcomes (as measured by SHAPS).^15^

In our study, higher levels of anhedonia (as measured by SHAPS and DARS) were associated with greater presenteeism and non-work-related activities. Additionally, only 35% of respondents with MDD reported to be employed. Therefore, the impact on lost productivity could be possibly underrepresented in this study. A cross-sectional study by Johnston et al. reported that anhedonia negatively influenced both presenteeism and absenteeism.^33^ Nevertheless, analysis of individual depressive symptoms showed variations in terms of their impact on presenteeism and absenteeism. While cognitive symptoms such as impaired concentration, loss of pleasure, and self-criticism significantly impacted presenteeism they were less predictive of absenteeism.^33^ A large database study reported greater unemployment, presenteeism, absenteeism, and disability rates due to anhedonia in patients with MDD.^18^

Further, in this study, greater levels of anhedonia (as measured by SHAPS) were associated with increased total direct medical and office visit costs in the past 6 months. On the other hand, higher anhedonia (as measured DARS) was associated with more HCP and psychologist/therapist visits. Although prior research reported greater HCRU and substantial increase in total direct medical and indirect costs in patients with MDD,^5^^,^^6^ studies examining the economic burden of MDD due to anhedonia is scarce. While MDD has been associated with greater HCRU and higher costs, it is possible that those with MDD and higher anhedonia may have disproportionately greater medical costs compared to individuals with MDD and lower anhedonia.

While this study showed that anhedonia is linked to worse health outcomes, the present study did not investigate underlying mechanisms that may be causing this association. It is possible that shared underlying mechanisms, such as inflammation and chronic pain,^34^^,^^35^ may explain the relationship observed in this study. It is also possible that the relationship of anhedonia with health outcomes is mediated by another factor (e.g., loneliness)^36^ or that anhedonia acts as a mediator of the relationship between depression severity and health outcomes.^37^ More research, including longitudinal studies, is needed to better understand the causal pathway(s) linking anhedonia with poor health outcomes.

Although currently available antidepressants demonstrated improvement in symptoms of depression and occupational functioning, treatment of anhedonia remains challenging as there are few biological treatment options available that specifically target anhedonia.^8^^,^^9^ While there has been increasing interest in the development of treatment approaches, novel treatments targeting anhedonia symptoms that improve patients’ HRQoL and functioning are needed, particularly for patients experiencing partial or no response to current antidepressants. The overall findings of the study highlight a significant unmet need in the treatment of anhedonia and suggest the necessity for targeted treatments such as pharmacological therapies (dopaminergic agonists), behavioral or reward-based interventions (to increase the engagement of individuals), and neurostimulation approaches (like transcranial magnetic stimulation and electroconvulsive therapy by inducing changes in brain activity).^38^^,^^39^ These approaches may potentially address anhedonia as an important dimension of MDD, thereby improving clinical, humanistic, and economic outcomes for patients.

### Strength and Limitations

The study used NHWS population to identify respondents with MDD for participation in a recontact survey. This study used patient-reported outcome measures, which are not available via other data sources, such as claims databases or other electronic health record databases. Additionally, data was collected from a community-dwelling sample of adults diagnosed with depression rather than clinical settings. Our study also has certain limitations that need to be acknowledged. The study was limited to a small sample size of patients with MDD who completed the recontact survey. In addition, there could be potential bias between respondents who participated and did not participate in the recontact survey, as evidenced by the underrepresentation of younger adults, racial/ethnic minorities, and to a lesser extent, men when compared to the NHWS respondents meeting eligibility criteria for the recontact survey, who may experience different patterns of anhedonia deficits and related burdens. Use of patient-reported outcome measures potentially introduces inaccurate reporting and recall bias, and we were unable to verify the accuracy of data with an independent data source (e.g., electronic health records or clinician-administered assessments). The study is also limited by its cross-sectional nature as causal inferences cannot be drawn. Additionally, the study may likely underrepresent people without access to or comfort with the Internet, as well as less healthy elderly people, institutionalized patients, and those with severe comorbidities and disabilities, including those with higher levels of anhedonia (as evidenced by mean SHAPS and DARS scores being skewed towards lower anhedonia). Finally, in the present study, we did not control for overall depression severity while examining the association of anhedonia severity with health outcomes. This could be a direction for future research. However, our understanding is that depression severity may fall into the causal pathway between anhedonia and health outcomes. As such, controlling for overall depression severity would likely result in controlling for anhedonia. Future research may also examine whether overall depression severity acts as a moderator of the relationship between anhedonia and health outcomes.

## CONCLUSION

This study provides novel insights into the burden associated with anhedonia severity among respondents with MDD in the US. In adults with depression, higher levels of anhedonia were associated with greater clinical, humanistic, and economic burden in patients with MDD. These results highlight the need for targeted treatments to help patients with MDD who have prominent anhedonia attain improved clinical, humanistic, work productivity, and economic outcomes.

## Supplementary Material

IntJNP-25-0018_R1_Suppl_Tables_and_Figures_pyaf048

## Data Availability

The data underlying this article were provided by Oracle Life Sciences under license. Data will be shared on request to the corresponding author with permission of Oracle Life Sciences.
