# Supplementary material for: Association between anhedonia severity and clinical, humanistic, and economic outcomes among US adults with major depressive disorder
Source: Int J Neuropsychopharmacol. 2025 Jul 2;28(8):pyaf048. doi: 10.1093/ijnp/pyaf048 (PMC12319313; doi:10.1093/ijnp/pyaf048)
Supplement: IntJNP-25-0018_R1_Suppl_Tables_and_Figures_pyaf048 [file intjnp-25-0018_r1_suppl_tables_and_figures_pyaf048.pdf]

Association between anhedonia severity and clinical, humanistic, and economic outcomes among US adults with major depressive disorder

Authors:

Hrishikesh Kale, MS, PhD[1]; M. Janelle Cambron-Mellott, PhD[2]; Tiina Drissen, PhD[1]; Kacper Perkowski, MS[2]; Kristen King-Concialdi, BS,[2]; Manish K. Jha, MD[3]

Supplementary Tables:

Supplementary Table 1: Key sociodemographic characteristics of NHWS respondents meeting eligibility criteria for recontact survey compared to final analysis sample

Characteristic	Eligible NHWS Sample (N = 8610)	Final Analysis Sample (N = 665)
Gender, n (%)		
Male	2315 (26.0)	144 (21.7)
Female	6595 (74.0)	521 (78.3)
Age, years, mean (SD)	47.2 (17.5)	58.4 (13.4)
Age category, n (%)		
18 to <25	1307 (14.7)	20 (3.0)
25 to <35	1433 (16.1)	31 (4.7)
35 to <45	986 (11.1)	38 (5.7)
45 to <55	1520 (17.1)	114 (17.1)
55 to <65	2033 (22.8)	210 (31.6)
65 and older	1631 (18.3)	252 (37.9)
Race, n (%)		
White	6450 (72.4)	573 (86.2)
Black/African American	1195 (13.4)	50 (7.5)
Asian	310 (3.5)	11 (1.7)
Other	955 (10.7)	31 (4.7)
Ethnicity, n (%)		
Hispanic	1184 (13.3)	35 (5.3)
Non-Hispanic	7726 (86.7)	630 (94.7)
Marital status, n (%)		
Married/living with a partner	4026 (45.2)	304 (45.7)
Single/divorced/ separated/widowed	4859 (54.5)	361 (54.3)
Education, college degree or higher, n (%)	3485 (39.1)	249 (37.5)
PHQ-9 score (NHWS), mean (SD)	10.1 (6.7)	9.9 (6.2)

MDD, major depressive disorder; PHQ-9, 9-item Patient Health Questionnaire; SD, standard deviation

Supplementary Table 2: Correlation analysis of anhedonia measures to PHQ-9

	SHAPS			DARS		
**Variable**	***r***	***p***	**R^2**	***r***	***p***	**R^2**
PHQ-9 score*	0.24	<0.001	0.06	-0.31	<.001	0.10
(1) Anhedonia	0.25	<0.001	0.06	-0.33	<.001	0.11
(2) Depressed mood	0.25	<0.001	0.06	-0.30	<.001	0.09
(3) Sleep disturbance	0.06	0.156	0.00	-0.12	0.001	0.02
(4) Fatigue	0.19	<0.001	0.03	-0.26	<.001	0.07
(5) Appetite changes	0.12	0.003	0.01	-0.19	<.001	0.03
(6) Low self-esteem	0.21	<0.001	0.04	-0.24	<.001	0.06
(7) Concentration difficulties	0.20	<0.001	0.04	-0.22	<.001	0.05
(8) Psychomotor disturbances	0.09	0.027	0.01	-0.11	0.004	0.01
(9) Suicidal ideation	0.20	<0.001	0.04	-0.21	<.001	0.04

*PHQ-9 score completed during the recontact survey.

DARS, Dimensional Anhedonia Rating Scale; PHQ-9, 9-item Patient Health Questionnaire; r, Pearson correlation coefficient; R^2, effect size; SHAPS, Snaith-Hamilton Pleasure Scale.

Supplementary Table 3: Multivariable analyses of association of MDD-ANH severity with clinical, humanistic, and economic outcomes: DARS score

Outcomes	N	*β* (SE)	RR* (95% CI)	*p*-value	1-SD increase (13.45 points)	
					β	RR*
Screening tools†						
PHQ-9 score	665	-0.132 (0.017)	-	**<0.001**	-1.778	-
GAD-7 score	665	-0.056 (0.016)	-	**<0.001**	-0.757	-
HCRU in past 6 months‡						
HCP visits	665	-0.008 (0.003)	0.99 (0.99-0.10)	**0.002**	-0.112	0.89
ER visits	665	-0.008 (0.007)	0.98 (0.99-1.01)	0.240	-0.103	0.90
Hospitalizations	665	0.005 (0.009)	1.01 (0.99-1.02)	0.623	0.061	1.06
Psychiatrist visits	665	-0.001 (0.012)	0.999 (0.98-1.02)	0.908	-0.019	0.98
Psychologist/therapist visits	665	-0.032 (0.013)	0.97 (0.94-0.99)	**0.015**	-0.434	0.65
HRQoL†						
RAND MHC score	665	0.144 (0.031)	-	**<0.001**	1.933	-
RAND PHC score	665	0.043 (0.032)	-	0.171	0.582	-
EQ-5D Index score	665	0.002 (0.000)	-	**<0.001**	0.022	-
EQ VAS score	665	0.076 (0.074)	-	0.304	1.021	-
WPAI, (mean %)‡						
Absenteeism	209	0.004 (0.017)	1.00 (0.97-1.04)	0.833	0.049	1.05
Presenteeism	203	-0.005 (0.001)	0.995 (0.99-1.00)	**<0.001**	-0.067	0.94
Work Productivity Impairment	203	-0.004 (0.074)	0.996 (0.98-1.01)	0.560	-0.058	0.94
Activity Impairment	665	-0.004 (0.004)	0.996 (0.995-0.997)	**<0.001**	-0.055	0.95
Economic						
Total direct medical costs§	665	-0.005 (0.005)	0.995 (0.99-1.01)	0.265	-0.070	0.93
Office visit costs§	665	-0.010 (0.003)	0.99 (0.98-1.00)	**<0.001**	-0.141	0.87
ER visit costs§	665	0.008 (0.005)	1.01 (1.00-1.02)	0.092	0.109	1.12
Inpatient costs§	665	-0.006 (0.007)	0.99 (0.978-1.01)	0.439	-0.076	0.93

Note: controlled for age, sex, race, CCI, and insurance.

*exp(β) is not presented for HRQoL or economic items. exp(β), the rate ratio, is presented for all other items.

†GLM w/ Identity link; Interpretation: For each 1-point/1-SD increase in DARS score <the outcome> changes by an average of <β>, keeping other predictors constant.

‡GLM w/ Negative Binomial distribution; Interpretation: For each 1-point/1-SD increase in DARS score <the outcome> is <exp(β)> times higher, keeping other predictors constant.

§GLM w/ Log link; Interpretation: For each 1-point/1-SD increase in DARS score <the outcome> is exp(β)> times higher, keeping other predictors constant.

CCI, Charlson Comorbidity Index; CI, confidence interval; DARS, Dimensional Anhedonia Rating Scale; ER, emergency room; GAD-7, 7-item Generalized Anxiety Disorder Assessment; GLM, generalized linear models; HCP, healthcare provider; HCRU, healthcare resource utilization; HRQoL; health-related quality of life; MDD-ANH, major depressive disorder- anhedonia; MHC, Mental Health Composite; PHC, Physical Health Composite; PHQ-9, 9-item Patient Health Questionnaire; RR, rate ratio; SD, standard deviation; SE, standard error; SHAPS, Snaith-Hamilton Pleasure Scale; VAS, visual analog scale; WPAI, work productivity and activity impairment.

Supplementary Figures:

Supplementary Figure 1: Study design

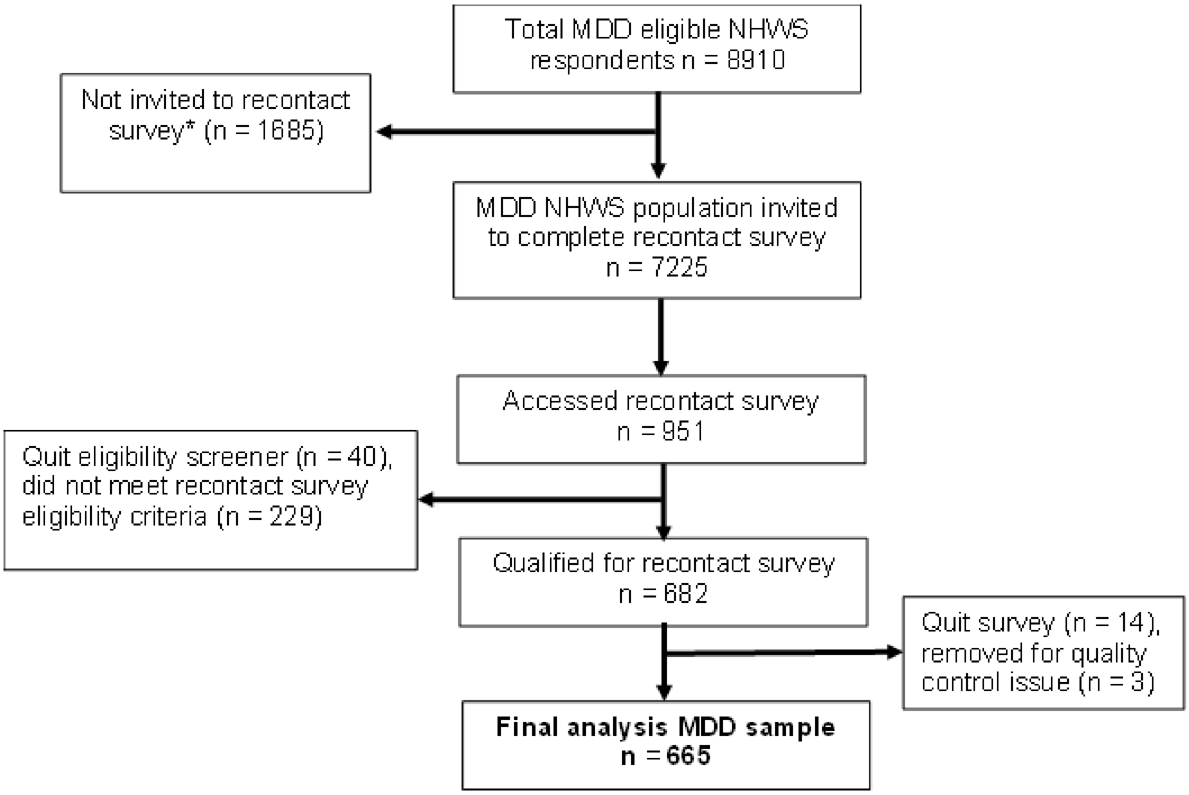

*Reasons for not being invited to survey include no longer being a member of the survey panel and panel company does not conduct recontact surveys.

MDD, major depression disorder; NHWS, National Health and Wellness Survey.

Supplementary Figure 2. Adjusted clinical outcomes by anhedonia severity: depression and anxiety

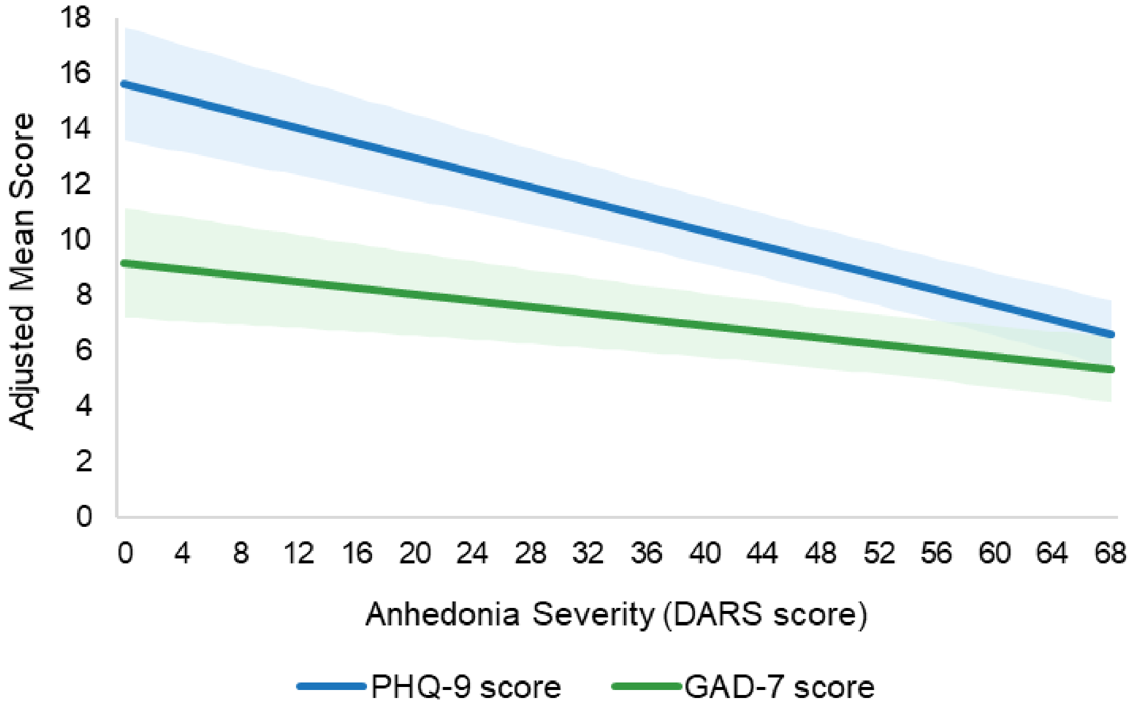

Note: Reference groups—age: 58.35 years, gender: male, race: White, insurance: commercial, CCI: 1.20. Lower DARS scores indicate greater anhedonia; shading represents 95% CI.

CCI, Charlson comorbidity index; CI, confidence interval; DARS, Dimensional Anhedonia Rating Scale; GAD-7, 7-item General Anxiety Disorder scale; PHQ-9, 9-item Patient Health Questionnaire.

Supplementary Figure 3. Adjusted humanistic outcomes by anhedonia severity:

HRQoL

A) RAND-36 MHC scores

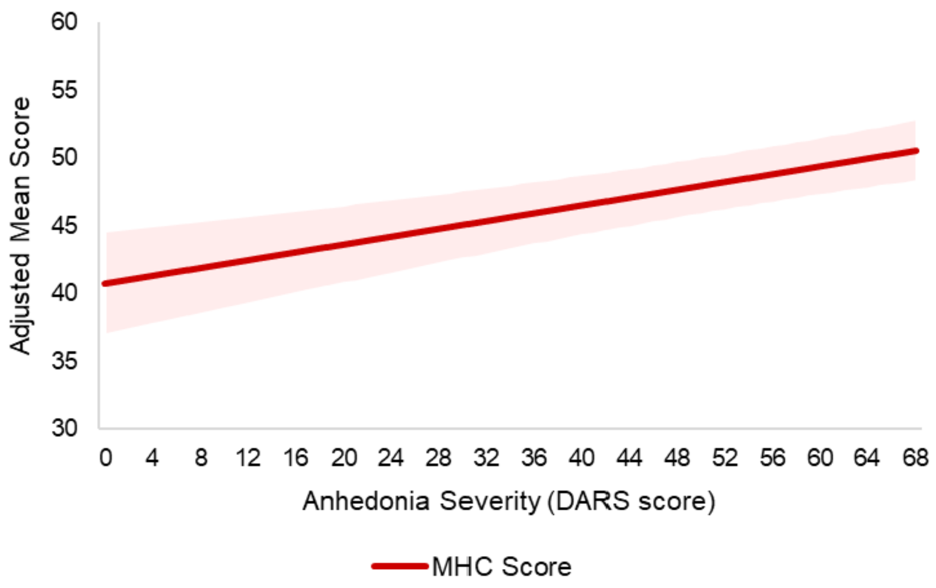

B) EQ-5D index scores

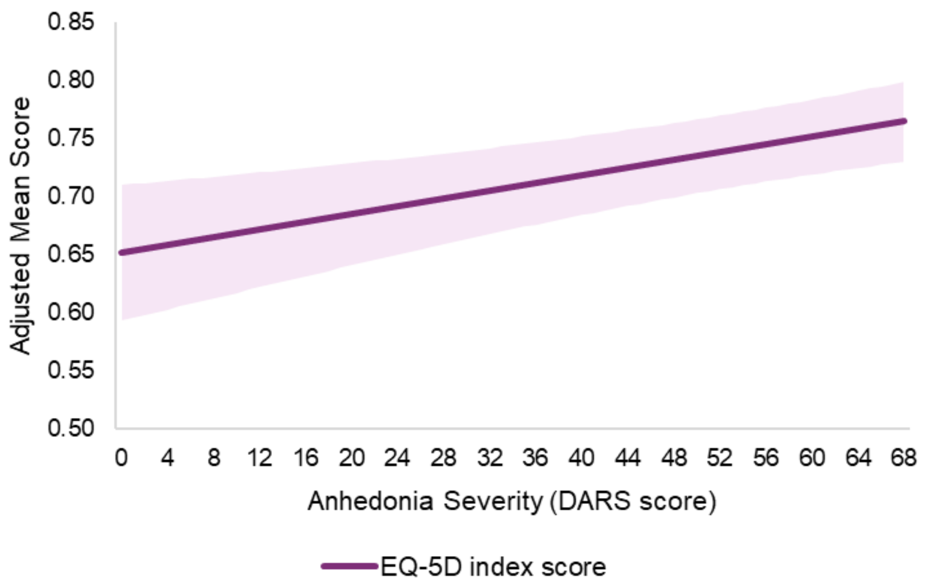

Note: Reference groups—age: 58.35 years, gender: male, race: White, insurance: commercial, CCI: 1.20. Lower DARS scores indicate greater anhedonia; shading represents 95% CI.

CCI, Charlson comorbidity index; CI, confidence interval; DARS, Dimensional Anhedonia Rating Scale; HRQoL, health-related quality of life; MHC, Mental Health Composite.

Supplementary Figure 4. Adjusted economic outcomes by anhedonia severity: WPAI

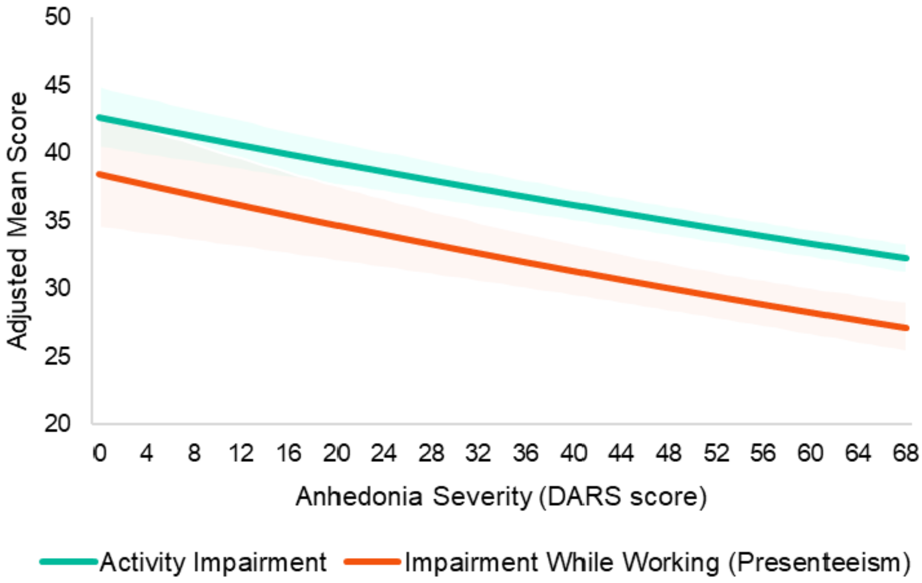

Note: Reference groups—age: 58.35 years, gender: male, race: White, insurance: commercial, CCI: 1.20. Lower DARS scores indicate greater anhedonia; shading represents 95% CI.

CCI, Charlson comorbidity index; CI, confidence interval; DARS, Dimensional Anhedonia Rating Scale; WPAI, work productivity and activity impairment.

Supplementary Figure 5. Adjusted economic outcomes by anhedonia severity: HCRU

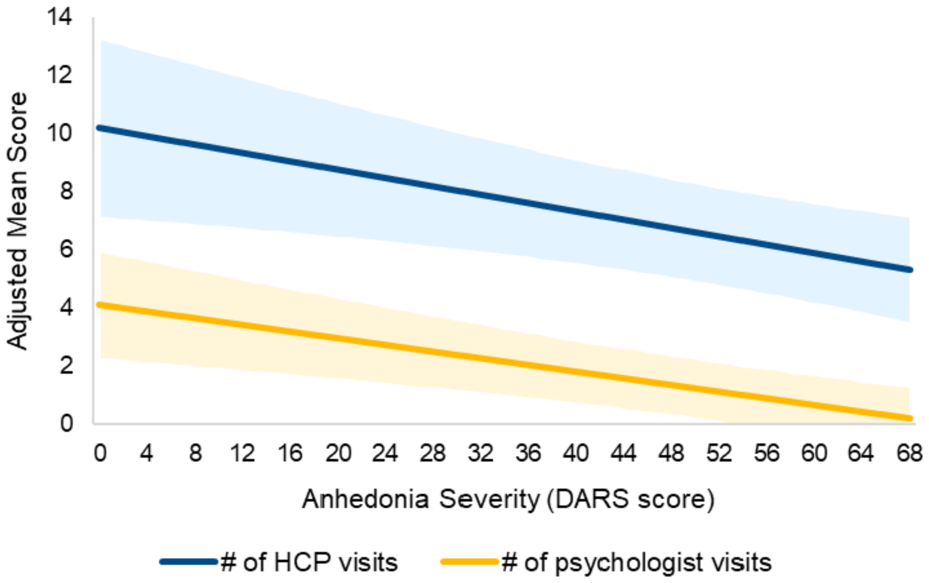

Note: Reference groups—age: 58.35 years, gender: male, race: White, insurance: commercial, CCI: 1.20. Lower DARS scores indicate greater anhedonia; shading represents 95% CI.

CCI, Charlson comorbidity index; CI, confidence interval; DARS, Dimensional Anhedonia Rating Scale; HCP, healthcare provider; HCRU, healthcare resource utilization.

Supplementary Figure 6. Adjusted economic outcomes by anhedonia severity: Office costs

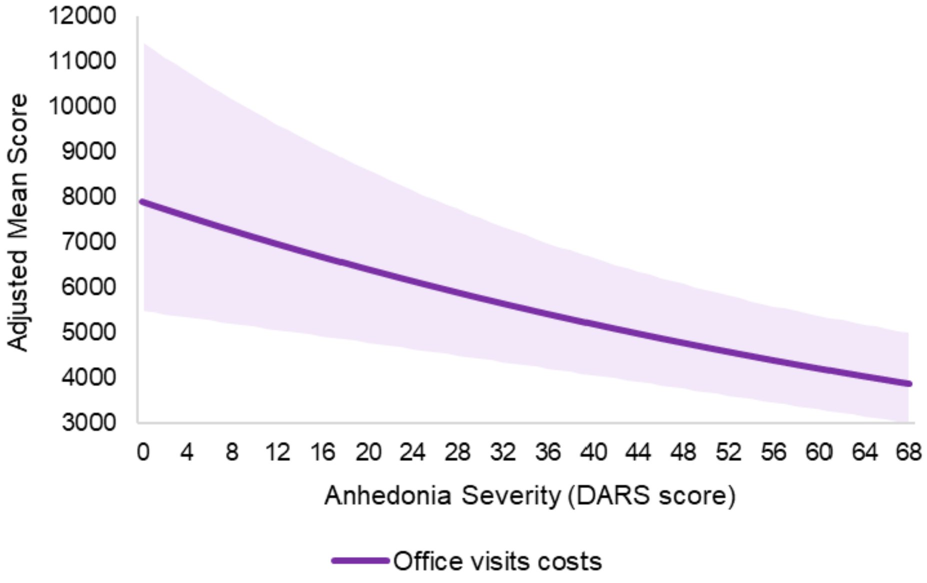

Note: Reference groups—age: 58.35 years, gender: male, race: White, insurance: commercial, CCI: 1.20. Lower DARS scores indicate greater anhedonia; shading represents 95% CI.

CCI, Charlson comorbidity index; CI, confidence interval; DARS, Dimensional Anhedonia Rating Scale.
